# Physical activity and gait in patients with idiopathic normal pressure hydrocephalus: a literature review

**DOI:** 10.3389/fneur.2025.1501709

**Published:** 2025-03-18

**Authors:** Kathrin Oebel, Tobias Moeller, Julie Etingold, Till Brombach, Sana Aslam, Justin L. Hoskin, Yonas E. Geda, Alexander Woll, Janina Krell-Roesch

**Affiliations:** ^1^Institute of Sports and Sports Science, Karlsruhe Institute of Technology, Karlsruhe, Germany; ^2^Department of Neurosurgery, Klinikum Karlsruhe, Karlsruhe, Germany; ^3^Department of Neurology, University of Colorado, Denver, CO, United States; ^4^Department of Neurology, Barrow Neurological Institute, Phoenix, AZ, United States

**Keywords:** idiopathic normal pressure hydrocephalus, CSF shunt surgery, gait, physical activity, dilated cerebral ventricles, Hakim’s triad

## Abstract

**Background:**

Idiopathic normal pressure hydrocephalus (iNPH) is characterized by impaired gait and cognition, and urinary incontinence. Even though iNPH still lacks standardized diagnostic criteria, many patients may potentially benefit from treatment which are often invasive procedures.

**Objectives:**

To provide an overview of the current state of research on physical activity behavior and gait in patients with iNPH, and examine potential changes after treatment (i.e., shunt surgery, spinal tap test or lumbar drainage).

**Methods:**

This literature review was carried out based on the PRISMA statement and we searched PubMed, Web of Science and Scopus databases in April 2023.

**Results:**

In total, 32 studies were included: 29 focusing on gait, 2 focusing on gait and physical activity, and 1 focusing on physical activity. All studies reported improvements in gait, such as reduced gait ataxia or shuffling gait and greater variability of gait cycle length, after an intervention or treatment. Improvements may depend on patients’ age, symptom duration, and treatment method, among others.

**Conclusion:**

Improvements in gait after iNPH treatment (e.g., shunt surgery) are well documented, whereas results on physical activity behavior in iNPH patients are inconsistent. More research on physical activity and gait outcomes before and after treatment is needed, also with regard to treatment success.

## 1 Introduction

Idiopathic normal pressure hydrocephalus (iNPH) is a chronic disease with constant progression of clinical symptoms that may impact quality of life (1). On brain imaging, patients exhibit dilated cerebral ventricles caused by a disturbance of cerebrospinal fluid (CSF) circulation, resulting in the so-called Hakim’s triad, which is characterized by gait abnormality, cognitive impairment, as well as bladder and/or fecal incontinence (2). INPH patients have a high prevalence of medical comorbidities, and a reliable diagnosis is often challenging particularly in the absence of standardized diagnostic criteria and treatment guidelines (2), albeit few guidelines have been proposed by various groups (3–6). Usually, iNPH is diagnosed based on a lumbar puncture (spinal tap test, TT), or placement of a lumbar CSF drainage to simulate a CSF shunt and corroborated by an examination of cognitive functions, assessment of potential incontinence, and gait testing (7); and treated through the implementation of a shunt system to drain excess fluid from the brain. INPH is still poorly studied, and is even met with some caution in the medical community (8). Thus, many affected individuals may not receive treatment, which may increase the need for care and mortality risk (9). Timely treatment based on a reliable diagnosis and continuous examinations after shunt surgery is warranted (9).

A majority of patients observe improvements in gait, along with improvement of other symptoms such as cognitive impairment, at 1-year post operation follow-up (10), and there is growing interest in better understanding gait and mobility in iNPH patients. However, gait assessments do not necessarily provide information about actual changes in patients’ everyday motor capacity, physical activity or sedentary behavior (11, 12). Regular physical activity is associated with better motor performance, higher functional independence, and improved physical and mental functioning in older adults. Therefore, in addition to gait, research on physical activity (behavior) in iNPH patients is important, but only few studies have been conducted on this (12, 13). Furthermore, while prior literature reviews and meta-analyses on iNPH have focused on the outcomes of pathogenesis, pathophysiology, key symptoms, radiologic findings, diagnosis, treatment or shunt responsiveness, to the best of our knowledge, none has considered physical activity or gait-related variables. Therefore, the aim of this literature review was to provide an overview of the current state of research on physical activity and gait in patients with iNPH. Specifically, we focused on studies that examined: (1) physical activity behavior in iNPH patients; (2) the impact of treatment (i.e., CSF shunt surgery, TT or lumbar CSF drainage) on physical activity, and preferably provide a comparison of physical activity before and after treatment; (3) gait in iNPH patients; (4) the impact of treatment on gait, and preferably provide a comparison of gait before and after treatment. Other commonly reported treatment outcomes of iNPH, such as cognitive impairment, were not emphasized in this review, and the focus was deliberately placed on physical activity and gait. We anticipate that this review will provide valuable insight into physical activity and gait in iNPH, and provide answers to the questions as to whether treatment may have an impact on these parameters in iNPH patients. The results of our review may thus have implications for clinical practice and may be of value to clinicians and researchers working with iNPH patients.

## 2 Materials and methods

We conducted a literature review based on the PRISMA Statement (14). Inclusion criteria was defined using the PICO scheme (15):

1. Population: Studies in patients with iNPH. We did not apply any further exclusion criteria regarding study population or disease status.
2. Intervention: Studies in patients who underwent CSF shunt surgery, TT or lumbar CSF drainage for iNPH treatment.
3. Comparison and Outcomes: Studies that examined physical activity and/ or gait (and gait-related parameters) before and/ or after CSF shunt surgery, TT or lumbar CSF drainage, respectively.

We only considered manuscripts published in English or German before April 11, 2023. The search terms consisted of combinations of three main keywords, i.e., idiopathic normal pressure hydrocephalus/ iNPH, physical activity, and gait. We searched PubMed, Scopus, and Web of Science databases. Please refer to Table 1 for the full search terms.

**Table 1:** Search terms by database.

Database	Search terms
PubMed	(“iNPH”[Title/Abstract] OR “normal pressure hydrocephalus”[Title/Abstract] OR “NPH”[Title/Abstract]) AND (“physical activit*”[Title/Abstract] OR “exercise”[Title/Abstract] OR “mobility”[Title/Abstract] OR “training”[Title/Abstract] OR “physical performance*”[Title/Abstract] OR “physical function*”[Title/Abstract] OR “movement”[Title/Abstract] OR “muscle activit*”[Title/Abstract] OR “gait”[Title/Abstract] OR “step”[Title/Abstract] OR “stride”[Title/Abstract] OR “walk*”[Title/Abstract])
Scopus	TITLE-ABS-KEY (“normal pressure hydrocephalus” OR “NPH”) AND TITLE-ABS-KEY (“physical activit*” OR exercise OR mobility OR training OR “physical performance” OR “physical function” OR movement OR “muscle activity” OR “gait” OR “step” OR “stride” OR “walk”)
Web of Science	[TS = (“normal pressure hydrocephalus”) OR TS = (NPH)] AND [TS = (“physical activit*”) OR TS = (exercise) OR TS = (mobility) OR TS = (training) OR TS = (“physical performance*”) OR TS = (“physical function”) OR TS = (movement) OR TS = (“muscle activit*”) OR TS = (gait) OR TS = (step) OR TS = (stride) OR TS = (walk*)]

* was used to search databases for variable endings of the root word.

## 3 Study selection and data extraction

After removing duplicates, all relevant publications were checked for eligibility by screening titles, abstracts, and finally, the full text, and included if they met the aforementioned inclusion criteria. Zotero software (version 6.0.26) was used for literature management. Relevant data from included studies were extracted to Microsoft Excel (version 16.16.27).

## 4 Assessment of methodological quality

The Critical Appraisal Skills Program (CASP) (16) was used to determine the quality of included studies. Checklists provided by CASP contain questions to be answered with “Yes,” “No” or “Uncertain” as well as open ended questions. For our review, we rated questions that were answered with “Yes” as positive, questions answered with “No” as negative, and questions that could not be answered as neutral. The open questions were also categorized as positive or negative after being answered. Publications were considered to be of lower quality if more than half of the questions were answered negatively or received neutral rating, and of good quality if more than half of the questions were answered positively. Studies on which 10 or more questions were answered positively were rated as having very good quality.

## 5 Results

**Figure 1:**
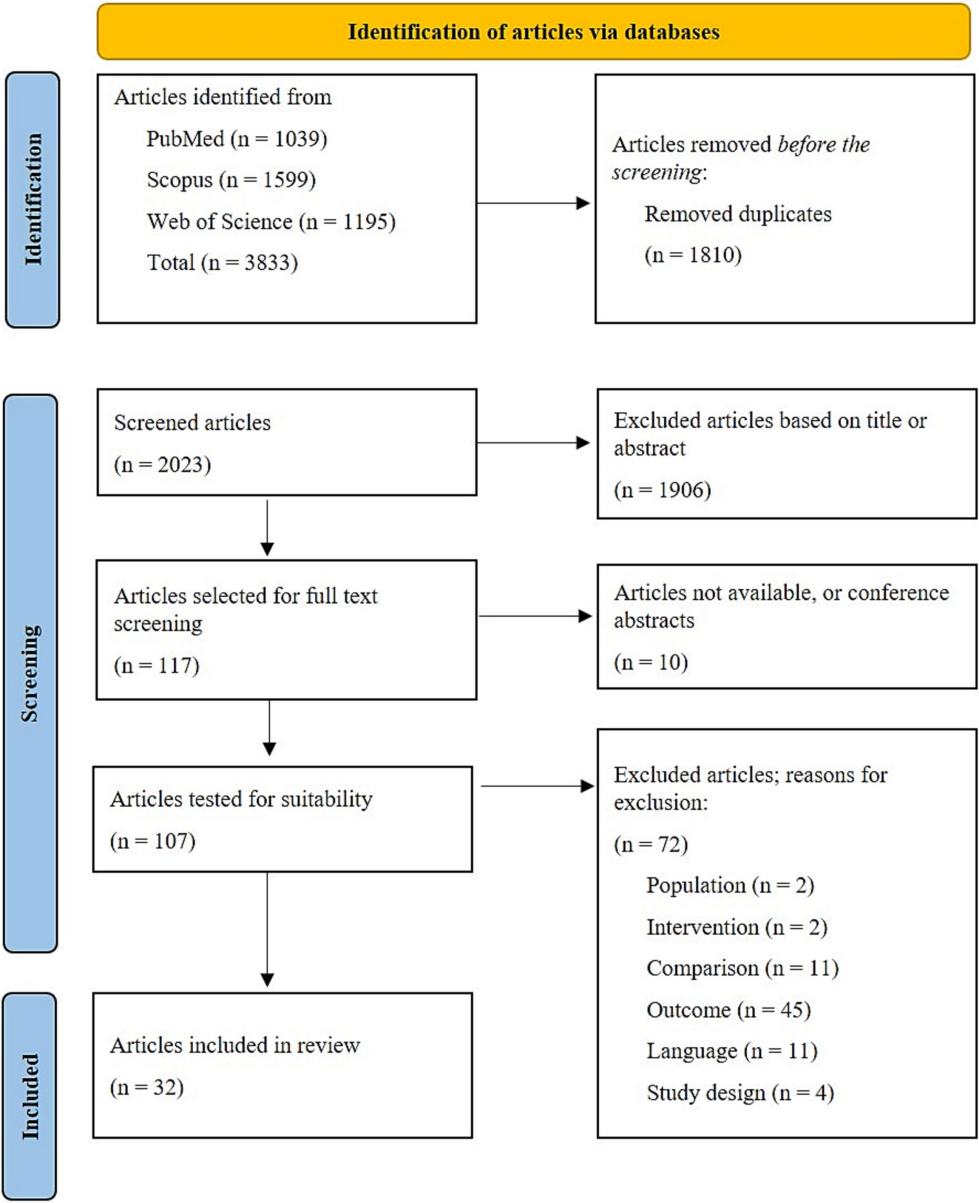
Flowchart for literature review (prepared based on PRISMA guidelines).

The search yielded a total of 3,833 publications, of which 32 were finally included in the literature review. For graphical display of the study selection process, please refer to Figure 1. A summary of included publications focusing on the outcome of physical activity is provided in Table 2, and on the outcome of gait in Table 3. An overview of gait parameters reported in included studies is provided in Table 4.

**Table 2:** Overview of included publications on the outcome of physical activity.

Author (Year)	Study population (n), sex (m/f), age (SD)	Intervention	Measurement system	Movement task	Results at follow-up (as compared to baseline)
Lundin et al. (11)	33, (17/16), 73 (49–81)	Shunt surgery	SenseWear bracelet (BodyMedia Inc., Pittsburgh, PA, USA)	-	No changes in steps/min, TEE, lying time, sleep duration after shunt surgery
Rydja et al. (12)	95, (56/39), 73.7 (6.6)	Shunt surgery	SenseWear actigraph (BodyMedia, Inc., Pittsburg, PA, USA)	-	No differences in PA, TEE and MET; ▲ voluntary walking (steps/min); share of night sleep
Sprau et al. (17)	2, (2/0), divided in “Patient 1″, 76; “Patient 2″, 70	Shunt surgery	Smartphone accelerometer, data exported from Apple Health via “QS Access” app (Quantified Self Labs, San Francisco, CA)	-	Patient 1: ▲ activity level (short-term), then ▼ from the sixth month after shunt surgery; Patient 2: ▲ activity level (sustainable)

PA, Physical activity; TT, Tap Test; LD, Lumbar drainage; m, male; w, female; ▲, increase; ▼, reduction; TEE, Total Energy Expenditure; MET, Metabolic Equivalent of Task.

**Table 3:** Overview of included publications on the outcome of gait.

Author (Year)	Study population (n), sex (m/f), age (SD or range)	Intervention	Measurement system	Movement task	Results at follow-up (as compared to baseline)
Agerskov et al. (43)	429, (266/163), 71 (9.5)	Shunt surgery	-	10MWT, TUG	▲ percentage share of patients able to walk independently (without walking aid), probability for only one type of gait disturbance; ▼ prevalence of gait abnormalities, for 180° rotation required steps
Agostini et al. (18)	60, (44/16), 73 (8)	TT, shunt surgery	STEP32 (Demitalia, Medical Technology, Italy), foot switches, knee goniometer	Walking 2.5 m on a 9 m long walkway, barefoot, self-chosen gait velocity	▼ gait impairment in all TT responders who had surgery, ▲ gait velocity in about half of the responders and one third of the non-responders
Armand et al. (1)	18, (6/12), 76 (7.4)	TT	Foot switches (AURION ZeroWire, Milan, Italy), optoelectronic camera system (VICON Mx3+, Vicon Motion Systems, Oxford, UK)	Walking with self-selected gait velocity on 10 m long walkway, single and dual task conditions (counting tasks)	Single task condition: No improvement; Double task condition: ▲ gait velocity, step length
Baltateanu et al. (26)	19, (12/7), 69.5 (66–77)	Shunt surgery	-	TUG, 10MWT	▼ TUG-time, ▲ 10MWT-velocity
Bovonsunthonchai et al. (39)	27, (16/11), 77.30 (60–89)	TT	Force Distribution Measurement Platform (FDM), video camera	TUG	▼ Sit-to-stand-time, gait duration, steps required for 180° turn; no differences in duration of the turn
Fraser & Fraser (27)	1, (1/0), 78 (0)	TT, shunt surgery	-	-	TT: Temporary improvement in gait, noticeable 3 h after TT for approx. 18 h; Shunt surgery: 3 months: stable gait, safe walking without walking aid; 6 moths: Improved gait, no falls; 1 year: further improvement of gait
Gago et al. (28)	8, (4/4), divided into “Minor improvement,” 74 (70/78); “Major Improvement,” 73 (59/81) after TT	TT, shunt surgery	Physilog® Sensors (GaitUp®, Switzerland)	20 m continuous walk: 10 m-long walk with 180°-turn, self-selected gait velocity	“Minor improvement”: Minor gait improvement of 10.2% after TT, no additional benefit after shunt surgery; “Greater improvement”: Gait improvements of 23.1% after TT, 82.9% after shunt surgery
Gallagher et al. (35)	74, (47/27), 75 (66.80; median) divided into “Responders” and “Non-responders”	TT	-	BBS, Tinetti (Tinetti, Tinetti Gait, Tinetti Balance), TUG; 10MWT	“Responders”: ▲ Tinetti balance, Tinetti gait, Tinetti, 10MWT, BBS; ▼ TUG; “Non-responders”: ▲ Tinetti gait, Tinetti, BBS
He et al. (29)	36 (27/9), divided into “Responders” 83.5 (5.8); “Non-Responders,” 69.6 (5.9)	LD	“Ambulatory Parkinson’s Disease Monitoring” (APDM Inc., USA)	Two-minute gait test, self-selected gait velocity, walking forwards on a 7 m path, 3 repetitions, selection of the best passage	“Responders”: ▲ cadence, gait velocity, foot strike angle, step length, steps in turning; ▼ percentage of double support; “Non-responders”: no improvements
Ishii & Akiguchi (19)	2, (2/0), 79 and 66 (6.5)	Shunt surgery	-	TUG	Improvement of gait disturbances and TUG time in both patients
Jung et al. (20)	1, (1/0), 72 (0)	TT, shunt surgery	-	-	Improvements of all gait parameters after TT, further improvements in gait after shunt surgery
Kitade et al. (40)	12, (5/7), 76.3 (4.6)	Shunt surgery	VICON MX 10-camera motion analysis system (Vicon Peak, Oxford, UK)	10 m walking, self-selected gait velocity	▲ gait velocity, step length, cadence, hip extension angle during stance phase, hip flexion moment during pre-swing phase, absorption power of the hip joint during terminal stance phase, motion of knee joint in sagittal plane; no differences in max. flexion angles, propulsive force of the hip and ankle joints regained
Krauss et al. (30)	11, (−/−), −	Shunt surgery	Treadmill, force-measuring system	Walking at a self-selected gait velocity	▲ gait velocity, gait cycle length; no changes in cadence; joint angle displacements in the knee joint and ankle joint, near normal range
Lim et al. (21)	23, (11/12), 73.0 (7.0)	TT	GAITRite Portable Walkway and Gait Analysis System (GAITRite, CIR System, Havertown, PA)	Barefoot walking at self-selected gait velocity without walking aid, 4 trials	▲ gait velocity, gait cycle length, cadence; ▼ step width, gait cycle time, variability of gait cycle time, variability of gait cycle length
Lundin et al. (11)	33, (17/16), 73 (49–81)	Shunt surgery	-	10MWT, TUG	Improvement of gait
Matousek et al. (31)	10, (8/2), 64 (16)	TT, shunt surgery	Non-invasive optoelectronic measurement technique with infrared light (Qualisys AB, Göteborg, Sweden)	Posturo Locomotion Manual (PLM)	2-3 h after TT: ▼ movement time, PLM improvements; 3 months after shunt surgery: ▼ movement time, PLM improvements
Morel et al. (41)	77, (52/25), 76.1 (6.2)	TT	Optoelectronic system (Vicon Mx3+, Oxford Metrics, UK)	Walking on 10 m-long walkway, self-selected gait velocity	▲ gait velocity in all patients except those with parkinsonian gait; greater improvements in patients with frontal gait compared to other gait phenotypes
Nikaido et al. (32)	99, (64/35), 77.5 (5.5)	Shunt surgery	-	Functional Gait Assessment (FGA)	▼ TUG time, ▲ FGA score, improvements in gait and balance function
Ravdin et al. (42)	33, (21/12); divided into “Responders“, 79.5 (4.5); “Non-Responders“, 77.0 (8.15)	TT	-	Walking 10 m, 180° turn	No differences between the groups in mean number of steps; “Responders”: difference in mean number of steps for 180° turn; “Non-Responders”: no differences
Razay et al. (22)	18, (9/9), 76.4 (58–92)	Shunt surgery	-	Tinetti balance and gait tests, TUG, 10MWT	▼ TUG time, 10MWT time, ▲ independence after shunt surgery, mental and physical disabilities in patients without shunt surgery; younger age, shorter duration of symptoms associated with more favorable outcomes
Rydja et al. (12)	95, (56/39), 73.7 (6.6)	Shunt surgery	-	10MWT, 6MWT, 30sCST	Improved short-distance walking, ▲ functional exercise capacity, functional strength; not influenced by training program
Schniepp et al. (45)	24, (17/7), 76.1 (7.8)	TT	GAITRite Portable Walkway and Gait Analysis System (GAITRite, CIR System, Havertown, PA)	Walking at self-selected gait velocity, walking at max. gait velocity, 2 cognitive dual-task conditions	Delayed change in gait velocity during the first three days after TT: ▲ gait velocity for single and double tasks, ▲ gait velocity 24–48 h after TT (max.)
Shaw et al. (23)	45, (32/13), 77.1 (7.2) divided into “Responders” and “Non-Responders”	Shunt surgery	-	TUG, 8MWT, modified version of the “Figure-of-Eight Walk Test,” turning tasks	“Responders”: ▲ gait time of all movement tasks; “Non-responders”: no changes
Shrinivasan et al. (38)	3, (−/−), −	TT	“TEMPO 3.1” (University of Virginia, Charlottesville, VA)	10 m walk, 360° turn in both directions, return to starting point	▼ variance of the gait parameters
Song et al. (36)	28, (16/12), 75.18 (7.29)	TT, shunt surgery	ProtoKinetics Zeno Walkway (ProtoKinetics LLC, Havertown, PA)	5TSTS, 25 walkway steps, 360°-rotation, Tinetti, Romberg	▲ step length, gait cycle length, standing phase, swing phase, single support phase, gait velocity, gait duration; no differences in inside and outside foot angle, cadence, step width
Souza et al. (33)	25, (10/15), 76.2 (5.8)	TT	Video camera	10 m walk at self-selected gait velocity, turn, 10 m walk back to starting point	Gait velocity characteristic with greatest improvement after TT, followed by cadence, stride length, 180° turns, step height
Stolze et al. (24)	10, (6/4), 75.9 (6.3)	TT	Walkway, video camera, treadmill, infrared movement analysis system (Qualisys, Sävedalen, Sweden)	Walking at self-selected gait velocity	▲ gait cycle length, gait velocity, unchanged cadence (due to increased gait cycle length), gait cycles more regular, swing phase; ▼ stance phase, double support phase; no changes in step height and joint angle excursion; greatest improvement in gait velocity, followed by gait cycle length, double support phase, stance and swing phase
Sundström et al. (44)	51, (29/22), 76.4 (6.0)	Shunt surgery	-	TUG, 10MWT	▼ TUG time, 10MWT time, greater improvements in patients with longer pre-operative (worse) TUG times, greater improvements in younger patients than in groups with comparable pre-operational TUG times
Warnecke (25)	1, (0/1), 78 (0)	TT	Video camera	10 m walk with rolator at the fastest gait velocity possible, 180° turn	▲ gait velocity, gait cycle length; ▼ cadence, steps required for 180° turn
Williams et al. (34)	19, (14/5), divided in “Shunted,” 73 (8); “Unshunted,” 76 (2)	LD, shunt surgery	GAITRite Portable Walkway and Gait Analysis System (GAITRite, CIR System, Havertown, PA)	Walking over gait mat, four trials	“Shunted”: LD: ▲ gait velocity, cadence; ▼ double support phase; no improvements in gait cycle length, variance coefficient of gait cycle length, stride length, base of support, FAP; Shunt surgery: improvements in all gait variables; “Unshunted”: LD: no improvements
Wolfsegger et al. (37)	21, (12/9), divided in “Shunt,” 70 (66–79); “No shunt“, 70 (63–80)	Shunt surgery	Pressure measuring mobile insole system (T&T Medilogic®, Berlin-Schoenefeld, Germany); Motion Capture System “Simi Motion” (Simi Motion; Simi Reality Motion Systems GmbH, Unterschleissheim, Germany)	Walking 10 m at a self-selected gait velocity	“Shunt”: After TT and shunt surgery: improvements in gait variables; “No Shunt”: After TT: no improvements in gait variables

PA, Physical activity; 6/8/10MWT, 6/8/10 meter walking test; TUG, Timed “Up and Go” test; TT, Tap Test; LD, Lumbar drainage; BSS, Berg Balance Scale; PLM, Posturo Locomotion Manual; FGA, Functional Gait Assessment; m, male; f, female; ▲, increase; ▼, reduction; FAP, Functional Ambulation Profile; 30sCST, 30-s chair stand test.

**Table 4:** Overview of gait parameters reported in studies.

Gait parameter	Influence of iNPH	Numbers of studies indicating this
Step height	▼ (shuffling gait, frozen gait)	9
Step length	▼ (short steps)	8
Balance (in general)	▼ (impaired)	8
Step width	▲ (broad-based gait, gait ataxia)	6
Gait velocity	▼	5
Step length variability	▲	4
Toe out angle	▲	3
Turning	Difficulties in turning	3
Steps required for 180° turn	▲	2
Foot strike angle	▼	2
Percentage of double support	▲	2
Percentage of swing phase	▼	1
Percentage of stance phase	▲	1
Lifting of forefoot toward end of swing phase	▼	1
TUG-time	▲	1
Sit-to-stand-time	▲	1
Duration of turn around	▲	1
Cadence	▼	1
Coronal range of lumbar motion	▼	1
Trunk movements	Unsteady	1
Variability of gait parameters	▲	1
Variability of foot angles	▼	1
Max. extension of knee joint	▼	1

▲, increase; ▼, decrease.

### 5.1 Study characteristics

The included manuscripts were published between 1995 and 2022, with 20 published within the last 10 years (2013–2022). Twenty-six publications utilized a cohort study design (*n* = 6 retrospective, *n* = 20 prospective), and we also included five case studies and one randomized controlled trial. All studies were conducted in a clinical setting. Three studies also used a field setting, as measurements were carried out in the participants’ home environments through actigraphy (11, 12, 17).

The studies had a total of 1,315 participants (807 males, 497 females, 14 no information on sex) with suspected or confirmed iNPH. In all studies, iNPH was defined by the presence of the Hakim’s Triad, including gait disturbance, cognitive impairment and urinary incontinence. In most studies, clinical symptoms were associated with ventricular dilatation (*n* = 9) (11, 18–25), and normal CSF pressure (*n* = 10) (17, 26–34). One study described that there is no obstruction to CSF flow (35), whereas three others (12, 36, 37) reported disturbances in CSF dynamics. Most authors followed the international guidelines for iNPH diagnosis published in 2005 (3). Mean age of participants ranged between 60 and 84 years; only two studies included participants with a mean age < 70 years (26, 31); and two did not provide age information (30, 38). The studies either involved shunt surgery (*n* = 13), TT in addition to shunt surgery (*n* = 6), TT alone (*n* = 11), lumbar CSF drainage (*n* = 1) or lumbar CSF drainage plus shunt surgery (*n* = 1). Follow-up periods differed considerably across studies, and most studies (*n* = 14) did not have a long-term follow-up after the intervention, i.e., 1 hour to 1 week after the intervention (21, 24, 29, 32, 33, 35) or did not provide follow-up information (1, 25, 34, 38–42). Five studies had a 6 month follow-up period (12, 18, 28, 36, 43) and another six studies had a follow-up of three months (11, 22, 31, 37, 44) or 12 weeks (19) after shunt surgery, and a one-month follow-up was reported by two studies (20, 26). One study (30) had a follow-up of 11 weeks, two studies (23, 45) of 3–12 months, and two studies (17, 27) had a rather long follow-up period of 1 year after shunt surgery.

### 5.2 Physical activity in iNPH patients

Knowledge about the general physical activity level of patients with iNPH is limited (12). One study (11) stated that their sample was in the lower normal range in terms of number of steps per minute, but participants had a reduced total energy expenditure (TEE). There was no difference in lying time and sleep duration compared to healthy controls.

### 5.3 Physical activity in iNPH patients before and/ or after shunt surgery, TT or lumbar drainage

One case study (17) reported that one iNPH patient had a sustained increase and a second patient had only a short-term increase (less than 6 months) in physical activity level after shunt surgery. In contrast, a cohort study did not yield any significant changes in the number of steps, TEE, lying time or sleep duration after shunt surgery (11). In one study, an additional physical exercise program did not have any further effects on physical activity in iNPH patients after shunt surgery (12), and there were no significant changes in the number of steps, TEE and MET. However, participants had an increased proportion of voluntary walking (steps per minute) and nocturnal sleep at both three- and six-months follow-ups after shunt surgery, and the authors observed a weak correlation between walking short distances and voluntary walking.

### 5.4 Gait in iNPH patients

It is well known that iNPH patients tend to present with progressive gait impairments (1, 11, 12, 19, 21–23, 25, 26, 28, 30, 32, 33, 36, 37, 44), accompanied by motor delays and lower extremities muscular weakness (17). Gait ataxia, i.e., lack of coordination of muscle movements, can occur in patients with iNPH (38). Many iNPH patients suffer from a shuffling gait, which is often described as magnetic (18, 41, 43). In addition, significantly increased variance in stride length (20, 24, 38), as well as reduced cadence (24, 29) and gait speed (24, 27, 29, 31, 45) have been reported in iNPH patients. Freezing of gait may also occur during walking (24, 34, 43). Frequently described gait-related symptoms in iNPH patients include but are not limited to impaired balance (17, 24, 27, 29, 34, 40, 45), and increased track or stride width resulting in a wide-based gait (24, 27, 31, 40, 41, 43, 45). The latter can be accompanied by difficulties in turning around, which manifests in an increased number of steps and duration required for a 180° turn (27, 29, 34, 39). Movements of the trunk and upper limbs are relatively less impaired in iNPH patients, but transition movements from sitting to standing take longer (39). In addition, the maximum extension of the knee joint is significantly reduced in patients with iNPH (24). Based on careful clinical evaluation, a distinction can be made between different types of gait phenomena in relation to iNPH, such as normal, frontal, parkinsonian and other gait phenotypes (41).

### 5.5 Gait in iNPH patients before and/ or after shunt surgery, TT or lumbar drainage

Several studies included in this review reported reduced gait disturbance (e.g., gait ataxia or shuffling gait) in iNPH patients after shunt surgery (11, 18, 20, 27, 28, 32, 34, 36, 37, 39, 42, 43), as well as TT (20, 27, 28, 35). Significant improvements in gait variables have also been observed after lumbar drainage (34), albeit they may not be to the same extent as after shunt surgery (20, 27, 34). Due to improved gait after shunt surgery, many iNPH patients are able to walk without a walking aid (22, 27, 43). Younger age (22, 44), and shorter symptom duration (22) appear to be associated with more favorable gait outcomes after shunt surgery. One study (44) reported significantly poorer performance of women compared to men before and after shunt surgery in Timed Up and Go and 10-meter walking tests. However, there were no significant differences in improvement rates. Another study (40) revealed increases in hip extension angle during stance phase, in hip flexion moment during the pre-swing phase, of hip joint force during terminal stance phase, and of knee joint movement in the sagittal plane after shunt surgery. In addition, two studies (21, 24) reported improvements in the variability of gait cycle length. In another study (27), improvements in gait were already noticeable 3 hours after CSF drainage and lasted for 18 h. In contrast, another research (45) revealed maximal improvements between 24 to 48 h after TT in most participants. Studies showed that after TT, gait speed (24, 33, 42), gait cycle length and double support phase duration (24), stance and swing phase duration (24), the ability to turn around and the tendency to fall (33, 42), as well as cadence and step height (33) are most likely to improve. Regarding the different gait phenotypes in patients with iNPH, investigators (41) observed that gait speed improved significantly after TT in all patients except those with parkinsonian gait. In addition, patients with frontal gait showed greater improvements compared to patients with other gait phenotypes. After TT, LD or shunt surgery, reductions in steps required for a 180° turn have been reported by different authors (25, 29, 39, 42, 43), and there is also a reduction in the duration of transition movements from sitting to standing after TT (39).

### 5.6 Quality assessment

Four studies included in this review were rated as having low quality (12, 17, 20, 30). Nineteen studies were rated as having good quality (1, 11, 18, 19, 21, 24, 25, 27–29, 31, 33, 38–44), and nine studies were rated as having very good quality (7, 22, 26, 32, 34–37, 45). Of note, no effect sizes were mentioned in any of the case studies. Furthermore, with the exception of two studies (32, 37), no confidence intervals were given in the cohort studies. The results of the quality assessment are provided in Tables 5–7.

**Table 5:** Quality assessment of included case studies.

Case studies/Question	1	2	3	4	5	6	7	8	9	10	11	Total
Fraser & Fraser (27)	1	1	1	-	1	0	-	0	1	1	1	7
Ishii & Akiguchi (19)	1	1	1	-	1	1	-	0	1	1	1	8
Jung et al. (20)	0	-	-	-	1	1	-	-	1	-	1	4
Sprau et al. (17)	1	1	1	-	1	0	-	0	0	1	-	5
Warnecke et al. (25)	1	1	1	-	1	1	-	0	1	1	1	8

Prepared based on Critical Appraisal Skills Program checklist for case studies; 1 = positive answer, 0 = negative answer, − = unclear.

**Table 6:** Quality assessment of included randomized controlled trials.

Randomized controlled trials/Question	1	2	3	4	5	6	7	8	9	10	11	Total
Rydja et al. (12)	1	1	0	-	0	1	1	0	-	1	-	5

Prepared based on Critical Appraisal Skills Program checklist for randomized controlled trials; 1 = positive answer, 0 = negative answer, − = unclear.

**Table 7:** Quality assessment of included cohort studies.

Cohort studies/ Question	1	2	3	4	5	6	7	8	9	10	11	12	Total
Agerskov et al. (43)	1	1	1	-	-	1	1	-	1	1	1	1	9
Agostini et al. (18)	1	1	0	1	-	1	1	-	1	1	1	1	9
Armand et al. (1)	1	0	1	1	1	-	1	-	-	1	-	1	7
Baltateanu et al. (26)	1	1	1	1	-	1	1	-	1	1	1	1	10
Bovonsunthonchai et al. (39)	1	1	1	1	-	-	1	-	1	1	1	1	9
Gago et al. (28)	1	1	1	1	-	-	1	-	-	1	-	1	7
Gallagher et al. (35)	1	1	1	1	1	-	1	-	1	1	1	1	10
He et al. (29)	1	1	1	1	-	-	1	-	1	1	1	1	9
Kitade et al. (40)	1	1	1	1	1	-	1	-	1	1	-	1	9
Krauss et al. (30)	1	-	1	-	0	1	1	-	-	1	-	1	6
Lim et al. (21)	1	1	1	1	-	-	1	-	1	1	1	1	9
Lundin et al. (11)	1	1	1	1	-	1	1	-	-	1	-	1	8
Matousek et al. (31)	1	1	1	1	0	-	1	-	1	1	1	1	9
Morel et al. (41)	1	1	1	1	1	0	1	-	-	1	-	1	8
Nikaido et al. (32)	1	1	1	1	1	-	1	1	1	1	1	1	11
Ravdin et al. (42)	1	1	1	1	1	-	1	-	-	1	-	1	8
Razay et al. (22)	1	1	1	1	0	1	1	-	1	1	1	1	10
Schniepp et al. (45)	1	1	1	1	1	1	1	-	1	1	1	1	11
Shaw et al. (23)	1	1	1	1	1	1	1	-	1	1	1	1	11
Shrinivasan et al. (38)	1	-	1	1	0	0	1	-	-	1	1	1	7
Song et al. (36)	1	1	1	1	0	1	1	-	1	1	1	1	10
Souza et al. (33)	1	1	1	-	0	-	1	-	1	1	1	1	8
Stolze et al. (24)	1	1	1	1	0	-	1	-	1	1	1	1	9
Sundström et al. (44)	1	1	1	1	0	-	1	-	1	1	1	1	9
Williams et al. (34)	1	1	1	1	1	-	1	-	1	1	1	1	10
Wolfsegger et al. (37)	1	1	1	1	1	1	1	1	1	1	1	1	12

Prepared based on Critical Appraisal Skills Program checklist for cohort studies; 1 = positive answer, 0 = negative answer, − = unclear.

## 6 Discussion

We conducted a review of literature on physical activity and gait in iNPH, including before and after invasive procedures for iNPH. Other iNPH treatment outcomes such as cognitive impairment, bladder or fecal incontinence were not considered. The majority of included studies (29 of 32) focused primarily on gait, and only three publications examined the influence of iNPH and shunt surgery, TT or CSF drainage on physical activity. In addition, two of these three studies (12, 17) were rated as having poor quality. In light of the importance of physical activity on physical and mental functioning in older adults, more research on physical activity in iNPH patients is clearly needed. Preferably, physical activity should be assessed using objective technologies such as accelerometers or other (wearable) sensors in future research. The physical activity parameters used by two studies (11, 12), i.e., number of steps per minute, TEE and the difference in lying time and sleep duration, appear to be useful to quantify physical activity on an individual participant level and could be used in future research. However, both studies (11, 12) suggest that improved physical or motor performance after treatment does not directly lead to increased physical activity, albeit acknowledging that rehabilitation programs could improve gait capacity and therefore lead to increased physical activity (11).

It is well documented that most iNPH patients suffer from gait impairments (1, 11, 12, 19, 21–23, 25, 26, 28, 30, 32, 33, 36, 37, 44). Since these impairments slowly progress over time (17), continuous monitoring of patients with suspected iNPH is crucial in order to potentially counteract severe symptom progression and ensure preserved activities of daily living. In general, gait speed of iNPH patients is reduced due to shorter stride lengths caused by co-contractions of the proximal muscles and likely not due to reduced cadence (24, 45). The shortening of swing phases and the lengthening of stance phases also contribute to reduced gait speed. A broad-based, shuffling gait and freezing of gait have been described as the most frequently observed gait impairments (43). Therefore, both step width and height appear to be relevant for an iNPH diagnosis. In addition, affected individuals have difficulties with turns, and require an increased number of steps and duration to perform a 180° turn (27, 29, 34, 39, 42). These parameters may thus also be important with regard to iNPH diagnosis.

All studies included in this review reported gait improvements after a TT, LD or shunt surgery in iNPH patients, but this effect may particularly apply to patients who respond positively to a previously performed TT (18, 23, 29, 35, 42). It can thus be assumed that shunt surgery is only effective in treatment of gait impairments in these patients. As patients with frontal gait in particular benefit more from a TT than other gait phenotypes, this parameter should also be considered when deciding on surgery indication (41). Younger patients as compared to older with comparable gait impairments (22, 44), and those with shorter compared to longer duration of symptoms (22) seem to benefit more from shunt surgery. Early diagnosis and timely treatment may thus be essential to avoid an increased need for care, and early treatment could insure recovery to higher functional levels (44).

There is evidence that positive effects on gait of patients after TT, e.g., based on multiparametric gait analysis, are most clearly recognizable when walking at self-selected speed (33). Improvements in gait appear to be measurable with a delay, approximately 12–48 h after performing a TT (27, 30, 31, 45). Therefore, gait analysis should not be performed immediately after TT, but at a later point in order to recognize all potential effects on walking performance. Gait velocity is considered the gait parameter with the largest expected improvements after a TT (24, 33, 42), and gait velocity is the most frequently cited parameter to improve after TT, LD or shunt surgery (18, 21, 23–25, 29, 30, 34, 36, 40, 41, 45). Significant improvements in stride length have also been reported after TT and shunt surgery (1, 29, 36, 40). Thus, gait speed and stride length may be important parameters for pre- and post-operative monitoring of patients and regarding indication for surgery. As improvements in gait speed in iNPH patients could be due to an increase in stride length (30), the two parameters should be considered in conjunction with each other, and also with regard to changes in cadence.

In clinical practice, this could mean that a standardized test battery including gait analysis should take place before and 12–48 h after TT or LD. When simulating CSF shunting via lumbar drain, care should be taken to remove the drain with a sufficient interval before implementing the test battery so as not to negatively impact the outcome. Symptoms of iNPH may progress depending on shunt-valve type and programming postoperatively; therefore, it is advisable to subject the patient to regular testing, ideally using a monitoring system. This could lead to more individualized shunt-valve programming, to earlier detection of shunt malfunction, and to a more patient-tailored approach to surgical iNPH treatment. At the same time, such a system, if sensitive enough, could aid in better detection of individual iNPH symptoms before surgery. The clinical goal is to detect iNPH as early as possible, as early treatment correlates with better outcomes. Symptoms in the early stages of iNPH, however, may be mild and improvement after probatory CSF-shunting can be discreet and difficult to measure. In the future, physical activity may be included as an independent factor in the diagnosis and follow-up of iNPH patients. This may allow for additional information on the impact of physical activity in iNPH patients and for evaluating a possible response to CSF-shunting.

Also, in light of the current shortage of literature on physical activity and gait outcomes in iNPH patients, there are still many research gaps that need to be addressed in future studies, e.g., behavioral consequences apart from surgical interests albeit they are likely difficult to explore in the short-term. Also, consensus should be reached on psychometric instruments exploiting latent traits such as fear of falling and fatigue as well as assessment of more complex motor abilities (e.g., climbing stairs, using a cane, bathing), since current research is restricted by mainly focusing on gait.

Limitations of this review pertain to the study selection, data extraction and quality control which were carried out by only one person, and may thus increase risk of bias. However, a second author checked the selection of studies to enhance reliability and validity. In addition, some publications were excluded due to language (not English or German), albeit they may have been relevant. The CASP rating revealed that some included studies were of poor quality and may thus have limited significance. Furthermore, the CASP does not generally provide items for a scoring system, and we made adjustments to also compare the ratio of positively, negatively and neutrally rated questions. Nevertheless, since we included different types of study designs in our review, we regard the CASP as appropriate, albeit other quality assessment tools could have been used as well. In addition, there was heterogeneity between studies with regard to applied methodology such as diagnosis of iNPH, or follow-up periods after surgery (with periods ranging from as little as 1 to 4 hours to 1 year) which makes it challenging to draw conclusions and may limit the generalizability of our findings. Also, we lumped together both tap-test/ external lumbar drainage and post-shunt gait/ physical activity outcomes, which was mainly due to a lack of studies, and we did not provide detailed comparison and contrast on the findings and the relevance and mechanisms behind similar or differential responses. Future research should address the limitations and research gaps outlined in this review, as well as focus on establishing widely-accepted diagnostic guidelines and identifying measurement systems that are most suitable for iNPH diagnosis, thereby also allowing for subgroup and sensitivity analyses on physical activity and gait in iNPH.

## 7 Conclusion

This review provides an overview of the current status of research on physical activity and gait in iNPH patients. Studies showed that treatment in iNPH may have an effect on gait, albeit differential effects may exist between treatment forms (i.e., CSF shunt surgery, TT or lumbar CSF drainage). Relatively less research exists on physical activity in iNPH, or the impact of treatment on physical activity in iNPH patients. More research is needed to confirm our observations.
